# The association of fasting triglyceride variability with renal dysfunction and proteinuria in medical checkup participants

**DOI:** 10.1007/s10157-025-02640-9

**Published:** 2025-02-28

**Authors:** Natsumi Matsuoka-Uchiyama, Haruhito A. Uchida, Tomohiko Asakawa, Yoshimasa Sakurabu, Katsuyoshi Katayama, Shugo Okamoto, Yasuhiro Onishi, Keiko Tanaka, Hidemi Takeuchi, Rika Takemoto, Ryoko Umebayashi, Jun Wada

**Affiliations:** 1 Department of Nephrology, Rheumatology, Endocrinology and Metabolism, Faculty of Medicine, Dentistry and Pharmaceutical Sciences, Okayama University, Okayama, Japan; 2 Department of Chronic Kidney Disease and Cardiovascular Disease, Faculty of Medicine, Dentistry and Pharmaceutical Sciences, Okayama University, 2-5-1 Shikata-Cho, Okayama, 700-8558 Japan; 3 Ultrasound Diagnostics Center, Okayama University Hospital, Okayama, Japan

**Keywords:** eGFR decline, Proteinuria, Renal dysfunction, Triglyceride variability, Fasting triglyceride

## Abstract

**Background:**

The association between the variability of triglyceride (TG) and chronic kidney disease (CKD) progression remains unclear. We examined whether intraindividual variability in fasting TG was associated with the exacerbation of CKD.

**Methods:**

We conducted a retrospective and observational study. 18,339 participants, who went through medical checkups and had checked their estimated glomerular filtration rate (eGFR) and semi-quantitative proteinuria by urine dipstick every year since 2017 for 4 years were registered. Variability in fasting TG was determined using the standard deviation (SD), and maximum minus minimum difference (MMD) between 2017 and 2021. The primary end point for the analysis of eGFR decline was eGFR < 60 mL/min/1.73 m^2^. The secondary end point for the analysis of proteinuria was the incidence of proteinuria ≥ ( ±) by urine dipstick.

**Results:**

The renal survival was lower in the higher-SD, and higher-MMD groups than in the lower-SD, and lower-MMD groups, respectively (log-rank test *p* < 0.001, and < 0.001, respectively). Lower SD and lower MMD were significantly associated with renal survival in the adjusted model (hazard ratio (HR), 1.12; 95% confidence intervals (CI), 1.04–1.21, and HR, 1.13; 95% CI 1.05–1.23, respectively). The non-incidence of proteinuria was lower in the higher-SD, and higher-MMD groups than in the lower-SD, and lower-MMD groups, respectively (log-rank test *p* < 0.001 and < 0.001, respectively).

**Conclusion:**

Fasting TG variability was associated with CKD progression in participants who went through medical checkups.

**Supplementary Information:**

The online version contains supplementary material available at 10.1007/s10157-025-02640-9.

## Introduction

The number of chronic kidney disease (CKD) patients, who need renal replacement therapy, such as hemodialysis and kidney transplant has been increasing worldwide [1, 2]. Especially, CKD is one of the serious risk factors to lead to cardiovascular diseases and death. CKD has various risk factors, and especially hypertension and diabetes mellitus (DM) are the most established factors. In addition, it has been reported that the variability of blood pressure and blood glucose levels is also associated with renal dysfunction [3–6]. However, others are emerging, and yet unknown. Thus, identification and treatment of modifiable risk factors are the best ways to prevent and delay CKD development [7].

Hypertriglyceridemia has been implicated in the development and progression of renal damage [8–10]. Indeed, the abnormal deposition of lipids within the intrarenal vascular bed has been shown to contribute to glomerular injury by mechanisms involving increased oxidative stress and the production of proinflammatory cytokines as well as the hyperactivity of growth factors [11, 12].

We reported that postprandial triglyceride (TG) variability was suggested to be a risk factor for estimated glomerular filtration rate (eGFR) decline and the incidence of microalbuminuria in patients with type 2 DM [13]. Although TG is also one of the elements of metabolic syndrome and naturally fluctuates to a certain extent like blood pressure and blood glucose levels, there are only few reports about the association between the variability of TG and CKD progression and it remains unclear.

Therefore, the present study investigated the association of intraindividual variability in fasting TG with eGFR decline and the incidence of proteinuria in medical checkup participants, whose cardiovascular risk is quite low, to clarify whether fasting TG variability is associated with the exacerbation of CKD.

## Materials and methods

### Study design and participants

**Fig. 1:**
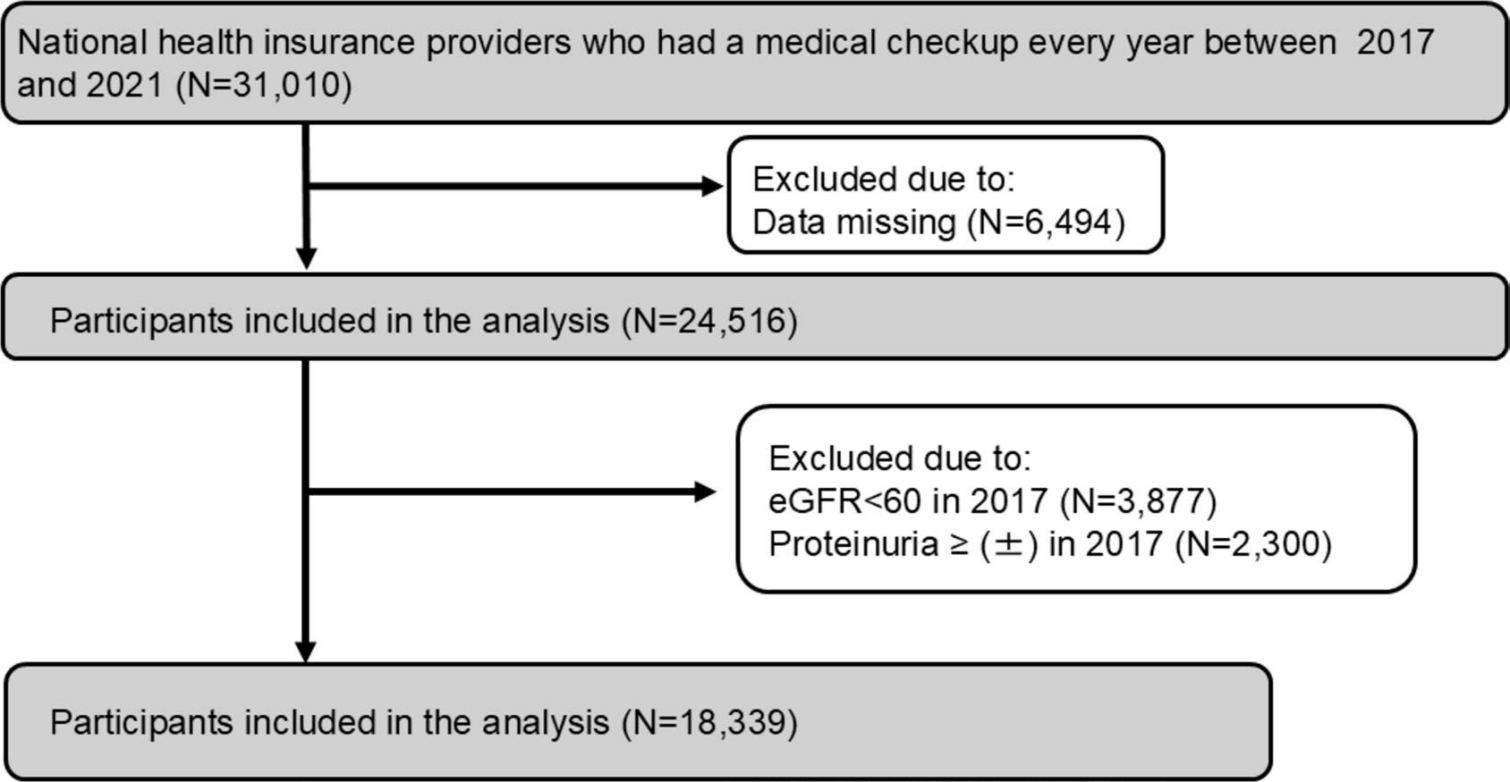
Flow diagram of the screening and enrollment of study participants. After excluding subjects who did not meet our study criteria, a total of 18,339 participants were included. eGFR, estimated glomerular filtration rate

A longitudinal and observational cohort study was conducted to examine the association of fasting TG variability with eGFR decline and the incidence of proteinuria. This study followed the Declaration of Helsinki on medical protocol and ethics. The ethics committees of Okayama University Hospital Institutional Review Board (accredited ISO9001/2000), Okayama, Japan, approved the protocol (approval number: 2104-006). We retrospectively reviewed National Health insurance providers in Okayama prefecture between 2017 and 2021 via electronic-based records. The participants were 40–75 years old, and went through medical checkups, including eGFR and semi-quantitative proteinuria by urine dipstick, every year since 2017 for 4 years. After excluding subjects whose eGFR was already less than 60 ml/min/1.73 m^2^ and/or whose proteinuria was already ( ±) or more in 2017, a total of 18,339 participants were included in the analysis (Fig. 1).

Two indices of fasting TG variability were calculated: the standard deviation (SD) and maximum minus minimum difference (MMD) of fasting TG between 2017 and 2021 in the same way as the previous papers [13–15]. The primary end point for the analysis of eGFR decline was when eGFR < 60 ml/min/1.73 m^2^ for the first time. The secondary end point for the analysis of proteinuria was when the incidence of proteinuria ≥ ( ±) by urine dipstick for the first time. Subjects who reached the end point were dropped from the analysis even if eGFR or proteinuria subsequently improved.

### Anthropometric measurements

The body mass index (BMI) was calculated as weight (kg) divided by height (m) squared.

### Laboratory measurements

The following patient characteristics were collected in 2017: age, sex, BMI, systolic and diastolic blood pressure, eGFR, serum creatinine (s-Cr), high-density lipoprotein cholesterol (HDL-C), low-density lipoprotein cholesterol (LDL-C), glycated hemoglobin (HbA1c), uric acid, smoking habit (current or not), quantitative measure of proteinuria, antilipidemic drug intake, and alcohol consumption (daily and occasionally, or never). eGFR was calculated using the formula modified for Japanese subjects: eGFR (mL/min/1.73 m^2^) = 194 × s-Cr (mg/dL)^−1.094^ × Age^−0.287^ (× 0.739 for females) [16]. Fasting status was defined as a fasting time between 8.0 and 23.9 h. The mean TG is the average fasting TG value between 2017 and 2021. Serum TG was assessed using an enzyme method (TG-EX, Denka®). The assay was performed within 24 h with an automated clinical chemistry analyzer.

### Definition of risk factors and covariates

CKD was defined as eGFR < 60 mL/min/1.73 m^2^. DM was defined as HbA1c ≥ 6.5%, a self-reported history of DM, and/or the use of any anti-diabetes medication. Hypertension was defined as systolic blood pressure (sBP) ≥ 140 mmHg and/or diastolic blood pressure (dBP) ≥ 90 mmHg, a self-reported history of hypertension, and/or the use of any antihypertensive drug. Regarding smoking status, current smokers were defined as participants who had a regular cigarette smoking habit in 2017. As for alcohol consumption, daily and occasional drinkers were defined as participants who had a habit of drinking alcohol daily or occasionally in 2017. Proteinuria was defined by urine dipstick tests using semi-quantitative measurements ≥  ± . Antilipidemic drug intake was defined based on the presence of a regular intake of such drugs in 2017.

The definition of risk factors for the multivariate Cox’s proportional hazard regression model was as follows: (1) age, BMI, baseline eGFR, mean TG as continuous variables, (2) sex: male, (3) proteinuria: urine dipstick tests by semi-quantitative measure ≥  ± , (4) smoking habit: current smoker, (5) DM: HbA1c ≥ 6.5%, a self-reported history of DM, and/or the use of any anti-diabetes medication, (6) hypertension: sBP ≥ 140 mmHg and/or dBP ≥ 90 mmHg, a self-reported history of hypertension, and/or the use of any antihypertensive drug, (7) antilipidemic drug intake: taking antilipidemic drugs in 2017, and (8) alcohol consumption: daily and occasional drinker.

### Statistical analyses

Data were expressed as n (%) for categorical variables and the median (interquartile range) for continuous variables. A Kaplan–Meier analysis and Cox’s proportional hazard regression model were adopted to calculate the cumulative probability to reach the end point and hazard ratio (HR) of eGFR decline and incidence of albuminuria. The estimated standard error of the confidence estimate was used to establish confidence intervals (CI) of the estimated HR. The statistical analyses were performed using the JMP software program, version 14.0.0 (SAS Institute, Inc, Cary, NC), and all *p* values were calculated as two-sided. The association was considered significant with *p* values less than 0.05.

## Results

### Characteristics

The baseline characteristics of the study participants for the analysis of eGFR decline, divided by the median SD and MMD values are listed in Table 1. The median SD and MMD values were 22 and 53, respectively. Compared with the lower-SD, and lower-MMD groups, the participants in the higher-SD and higher-MMD groups had higher BMI, sBP, dBP, s-Cr, LDL-C, HbA1c, and uric acid, lower eGFR and HDL-C, higher prevalence of DM, and hypertension, and more participants who smoked, drank alcohol, and received antilipidemic drug (Table 1).

**Table 1:** Characteristics of the study participants for the analysis with SD and MMD of the fasting TG variability groups (*n* = 18,339)

Variable	Higher-SD group: SD ≥ 22 (*n* = 9,383)	Lower-SD group: SD < 22 (*n* = 8,956)	*p* value	Higher-MMD group: MMD ≥ 53 (*n* = 9,061)	Lower-MMD group: SD < 53 (*n* = 9,278)	*p* value
Age (yr)	64 ± 7	64 ± 7	0.1745^*^	64 ± 7	64 ± 7	0.3942^*^
Sex (male)	3925 (44)	2840 (30)	< 0.0001^**^	4023 (43)	2742 (30)	< 0.0001^**^
BMI (kg/m^2^)	23.3 ± 3.2	22.0 ± 3.2	< 0.0001^*^	23.3 ± 3.2	22.0 ± 3.2	< 0.0001^*^
sBP (mmHg)	130 ± 17	127 ± 17	< 0.0001^*^	129 ± 17	127 ± 17	< 0.0001^*^
dBP(mmHg)	76 ± 11	75 ± 11	< 0.0001^*^	76 ± 11	75 ± 11	< 0.0001^*^
eGFR (mL/min/1.73m^2^)	76.2 ± 11.5	76.6 ± 12.0	0.0087^*^	76.1 ± 11.4	76.6 ± 12.1	0.0012^*^
s-Cr (mg/dL)	0.69 ± 0.13	0.67 ± 0.12	< 0.0001^*^	0.69 ± 0.13	0.66 ± 0.12	< 0.0001^*^
maximum TG (mg/dL)	210 ± 113	98 ± 31	< 0.0001^*^	207 ± 112	97 ± 30	< 0.0001^*^
minimum TG (mg/dL)	94 ± 43	66 ± 25	< 0.0001^*^	93 ± 43	65 ± 25	< 0.0001^*^
mean TG (mg/dL)	143 ± 65	81 ± 28	< 0.0001^*^	142 ± 64	81 ± 27	< 0.0001^*^
SD	47 ± 37	13 ± 5	< 0.0001^*^	46 ± 37	13 ± 5	< 0.0001^*^
MMD	116 ± 90	33 ± 12	< 0.0001^*^	114 ± 89	32 ± 12	< 0.0001^*^
HDL-C (mg/dL)	60 ± 15	70 ± 17	< 0.0001^*^	61 ± 15	70 ± 17	< 0.0001^*^
LDL-C (mg/dL)	127 ± 30	123 ± 28	< 0.0001^*^	127 ± 30	123 ± 28	< 0.0001^*^
HbA1c (%)	5.7 ± 0.6	5.6 ± 0.5	< 0.0001^*^	5.7 ± 0.6	5.6 ± 0.5	< 0.0001^*^
Uric acid (mg/dL)	5.2 ± 1.2	4.8 ± 1.1	< 0.0001^*^	5.2 ± 1.2	4.8 ± 1.1	< 0.0001^*^
Smoking (current)	1085 (12)	663 (7)	< 0.0001^**^	1104 (12)	644 (7)	< 0.0001^**^
DM	1561 (17)	1325 (14)	< 0.0001^**^	1628 (18)	1258 (14)	< 0.0001^**^
Hypertension	4699 (52)	4048 (43)	< 0.0001^**^	4855 (52)	3892 (43)	< 0.0001^**^
Antilipidemic drug intake	2430 (27)	2079 (22)	< 0.0001^**^	2533 (27)	1976 (22)	< 0.0001^**^
Alcohol consumption (daily and occasional)	3923 (44)	3740 (40)	< 0.0001^**^	4036 (44)	3627 (40)	< 0.0001^**^

*p* values less than 0.05 are in bold

*TG* Triglyceride, *SD* Standard deviation, *MMD* Maximum minus minimum difference, *BMI* Body mass index, *sBP* systolic blood pressure, *dBP* diastolic blood pressure, *eGFR* estimated glomerular filtration rate, *s-Cr* serum creatinine, *HDL-C* High-density lipoprotein cholesterol, *LDL-C* Low-density lipoprotein cholesterol, *HbA1c* glycated hemoglobin, *DM* diabetes mellitus, maximum TG the highest value of fasting TG between 2017 and 2021, minimum TG, the lowest value of fasting TG between 2017 and 2021

^*^Student’s *t* test **Pearson’s Chi-square test

Categoric variables are presented as *n* (%) and continuous data are represented as mean ± SD

### Clinical outcomes

Among the 18,339 total participants, 3534 (19%) reached the primary end point and 4274 (23%) reached the secondary end point.

**Fig. 2:**
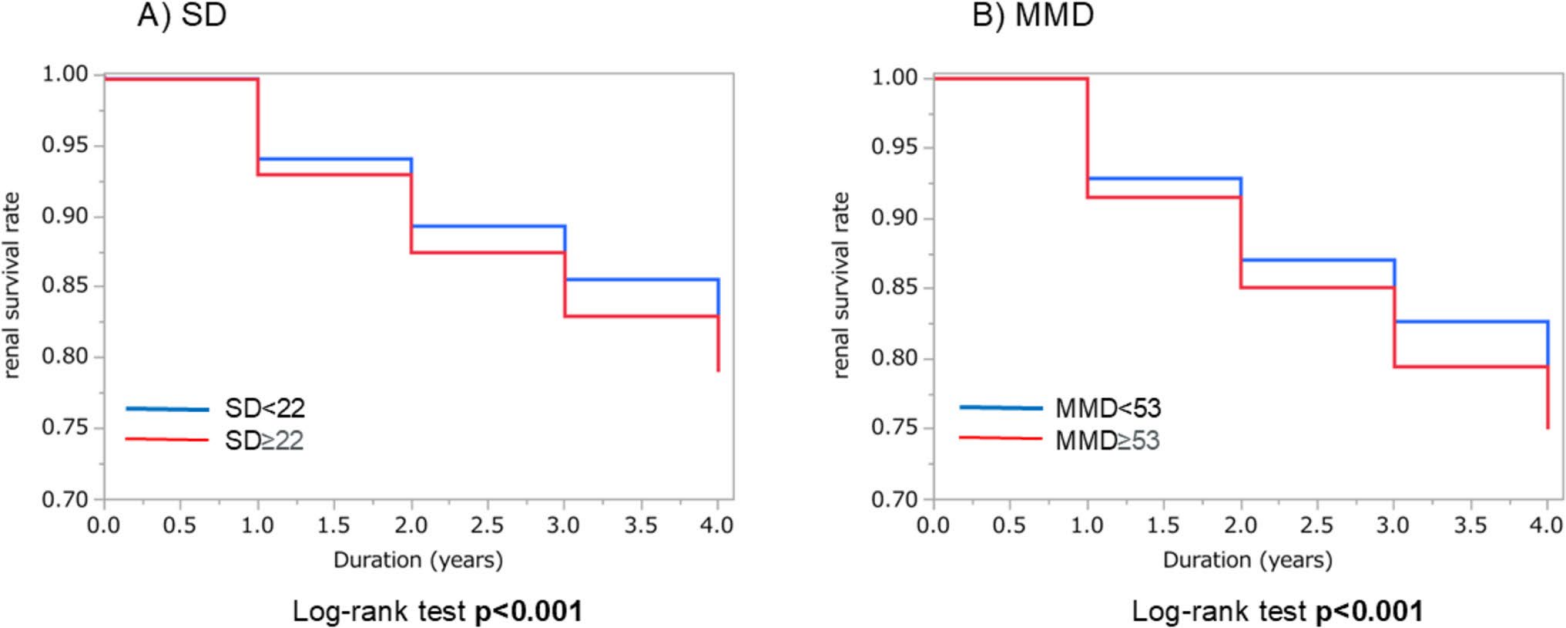
Comparing the renal survival rate between groups divided by the median values of SD and MMD. *SD* Standard deviation; *MMD* Maximum minus minimum difference

Regarding the primary outcome, the renal survival rate was 79% in the group with SD ≥ 22 and 82% in the group with SD < 22, while the renal survival rate was 79% in the group with MMD ≥ 53 and 83% in the group with MMD < 53. The renal survival rate was lower in the group with SD ≥ 22 than in the group with SD < 22 and lower in the group with MMD ≥ 53 than in the group with MMD < 53 (log-rank test *p* < 0.001 and *p* < 0.001, respectively) (Fig. 2). We performed a Cox’s proportional hazard regression analysis of the baseline factors for a possible association with renal survival. In this analysis, higher SD and higher MMD were significantly associated with the primary end point in the adjusted model (HR, 1.12; 95% CI 1.04 to 1.21 and HR, 1.13; 95% CI 1.05 to 1.23, respectively) (Table 2).

**Table 2:** Multivariate Cox’s proportional hazard regression model for the association between fasting TG variability and eGFR decline (primary end point)

HR [95% CI] *p* value	SD ≥ 22	MMD ≥ 53
Model 1	1.17 [1.10–1.26]** < 0.0001**	1.19 [1.12–1.28] < **0.0001**
Model 2	1.12 [1.04–1.21] **0.0045**	1.14 [1.06–1.24] **0.0007**
Model 3	1.12 [1.04–1.21] **0.0057**	1.14 [1.05–1.23] **0.0013**
Model 4	1.12 [1.04–1.21] **0.0033**	1.13 [1.05–1.23]** 0.0011**

Model 1: Adjusted for age, sex, and BMI

Model 2: Adjusted for age, sex, BMI, and mean TG

Model 3: Adjusted for age, sex, BMI, mean TG, and baseline eGFR

Model 4: Adjusted for age, sex, BMI, mean TG, baseline eGFR, smoking, DM, hypertension, antilipidemic drug intake, and alcohol consumption

*p* values less than 0.05 are in bold

*TG* triglyceride, *SD* standard deviation, *MMD* maximum minus minimum difference, *HR* hazard ratio, *95% CI* 95% confidence intervals, *BMI* body mass index, *eGFR* estimated glomerular filtration rate, *DM* Diabetes mellitus

**Fig. 3:**
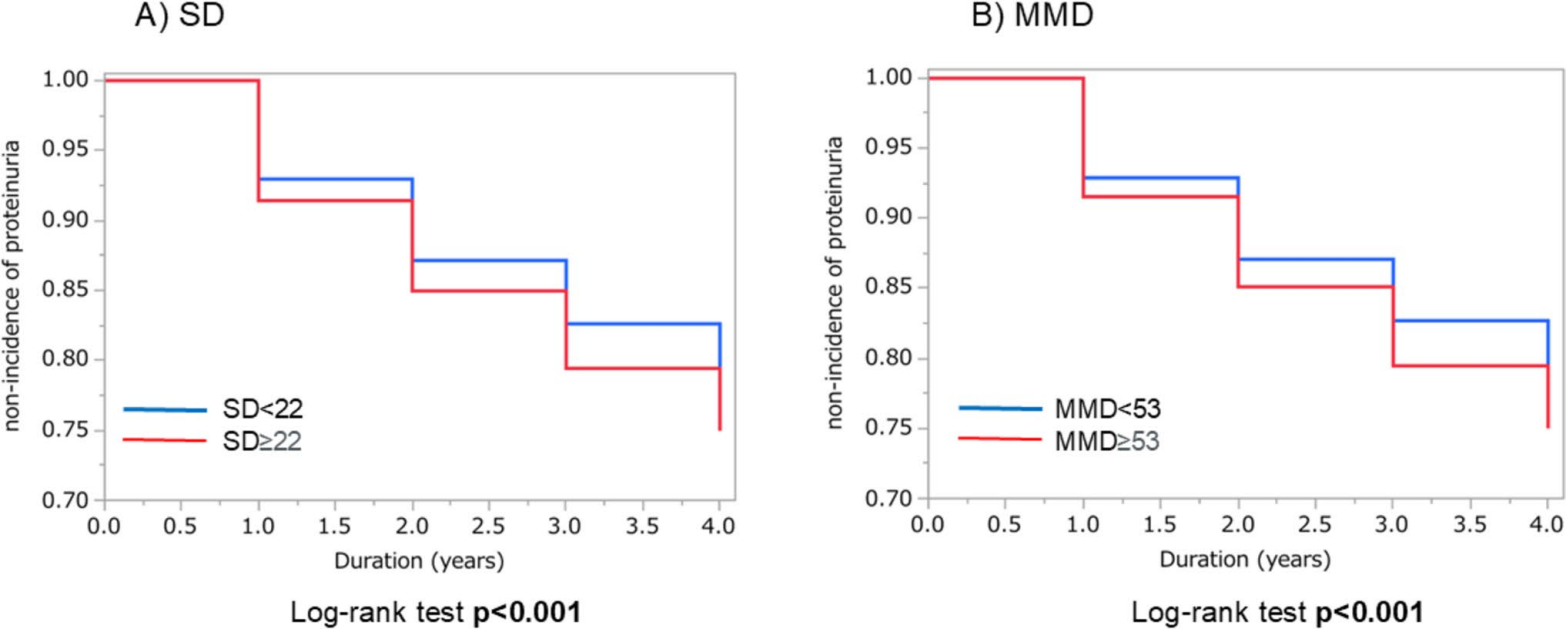
Comparing the non-incidence of proteinuria between groups divided by the median values of SD and MMD. *SD* Standard deviation; *MMD* Maximum minus minimum difference

Regarding the secondary outcome, the non-incidence of proteinuria was 75% both in the group with SD ≥ 22 and in the group with MMD ≥ 53, and 78% both in the group with SD < 22 and in the group with MMD < 53. The non-incidence of proteinuria was lower in the group with SD ≥ 22 than in the group with SD < 22 and lower in the group with MMD ≥ 53 than in the group with MMD < 53 (log-rank test *p* < 0.001 and *p* < 0.001, respectively) (Fig. 3). We performed a Cox’s proportional hazard regression analysis of the baseline factors for a possible association with the incidence of proteinuria. In this analysis, higher SD and higher MMD were significantly associated with the secondary end point in the adjusted Model 1 (HR, 1.08; 95% CI 1.01 to 1.15 and HR, 1.08; 95% CI 1.02 to 1.15, respectively) (Table 3). However, the significance of this association disappeared when further adjusted by mean TG, baseline eGFR, smoking, DM, hypertension, antilipidemic drug intake, and alcohol consumption (Model 2, 3, and 4) (Table 3).

**Table 3:** Multivariate Cox’s proportional hazard regression model for the association between fasting TG variability and the incidence of proteinuria (secondary end point)

HR [95% CI] *p* value	SD ≥ 22	MMD ≥ 53
Model 1	1.08 [1.01–1.15]** 0.0151**	1.08 [1.02–1.15]** 0.0129**
Model 2	1.02 [0.95–1.09] 0.6063	1.02 [0.95–1.10] 0.5361
Model 3	1.02 [0.95–1.09] 0.6087	1.02 [0.95–1.10] 0.5304
Model 4	1.01 [0.95–1.09] 0.6841	1.02 [0.95–1.09] 0.6082

Model 1: Adjusted for age, sex, and BMI

Model 2: Adjusted for age, sex, BMI, and mean TG

Model 3: Adjusted for age, sex, BMI, mean TG, and baseline eGFR

Model 4: Adjusted for age, sex, BMI, mean TG, baseline eGFR, smoking, DM, hypertension, antilipidemic drug intake, and alcohol consumption

*p* values less than 0.05 are in bold

*TG* Triglyceride, *SD* Standard deviation, *MMD* Maximum minus minimum difference, *HR* Hazard ratio, *95% CI* 95% Confidence intervals, *BMI* Body mass index, *eGFR* estimated glomerular filtration rate, *DM* Diabetes mellitus

**Fig. 4:**
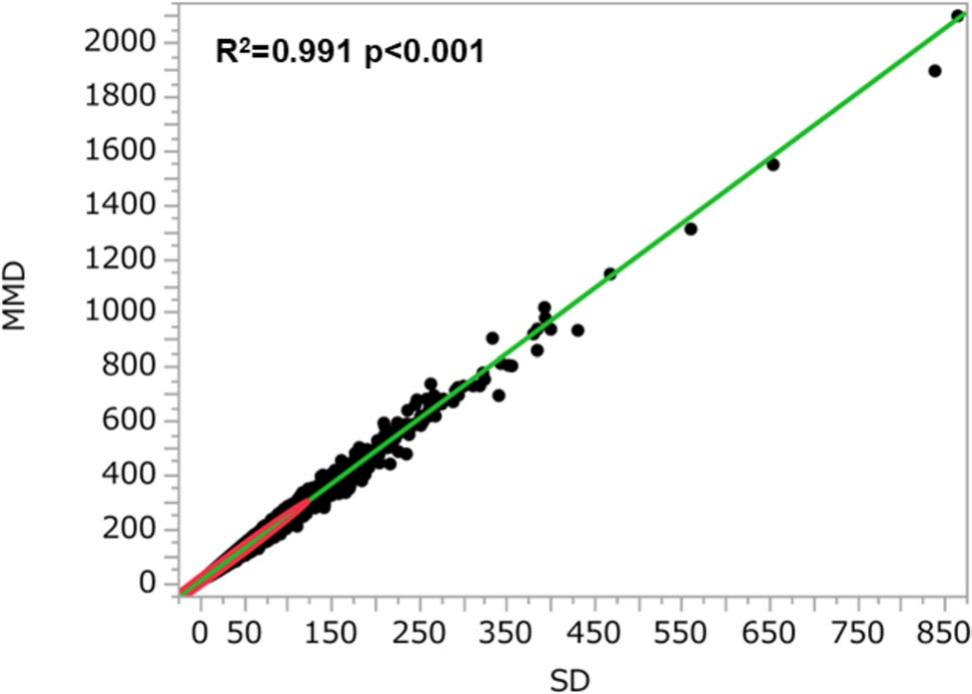
The association between SD and MMD. *SD* Standard deviation; *MMD* Maximum minus minimum difference

We conducted a scatter plot analysis with regression fit line representing the association between SD and MMD. There were very strong correlations between SD and MMD (*R*^*2*^ = 0.991; *p* < 0.0001) (Fig. 4). We also conducted scatter plot analysis with regression fit line representing the association between mean TG and fasting TG in 2017. There were very strong correlations between mean TG and fasting TG in 2017 (*R*^*2*^ = 0.676; *p* < 0.0001) (Supplementary Figure S1). However, the previous papers adopted mean TG as an adjusting factor to evaluate the variability instead of fasting TG itself and we followed it. However, when we adopt mean TG as an adjusting factor, it has a huge confounding effect. When mean TG is excluded from the adjusting factors, higher SD and higher MMD were significantly associated with the end point in most of the adjusted models (Supplementary Table 1S).

## Discussion

In this study, the association of fasting TG variability with the incidence of CKD and proteinuria in participants who took medical checkups by National Health insurance was examined. SD and MMD as the variability of fasting TG were significant predictors of eGFR decline, even when adjusting for confounding factors. In addition, the variability of fasting TG was also significantly associated with the incidence of proteinuria, although the significance of this association was diminished after adjusting for confounding factors. Thus, our study suggested that the variability of fasting TG was associated with CKD progression.

At molecular level, metabolic syndrome is characterized by a pro-inflammatory state and increased oxidative stress, leading to various pathophysiological changes causing endothelial dysfunction and a hypercoagulable state. Since kidney is a highly vascularized organ, it is especially susceptible to those microvascular changes related to eGFR decline and the incidence of proteinuria. Therefore, the metabolic syndrome and its individual components, such as hypertriglyceridemia, hyperglycemia, and hypertension, are associated with the premature development, acceleration, and progression of CKD [17].

Regarding "variability", it is reported that intermittent hyperglycemia is more detrimental to endothelial cells than continuous hyperglycemia [18]. In addition, not only hypertension, but also blood pressure variability is a significant prognostic factor in renal dysfunction [5, 6, 19]. Both the plasma glucose level and blood pressure naturally fluctuate to a certain extent and are components of metabolic syndrome, which are highly related to renal dysfunction. Given these factors, a similar association may be found for TG variability, which is another factor of metabolic syndrome and fluctuates to a certain extent. It has already been reported that fasting hypertriglyceridemia is associated with eGFR decline and incidence of albuminuria [20]. In addition, the variability of fasting TG is predictive of coronary events [15], and the variability of fasting TG is also linked to the incidence of microalbuminuria in patients with type 2 DM [14].

As for eGFR decline, it was suggested that the variability of fasting TG is associated with CKD progression, and it can be a predictor for eGFR decline. There were very strong correlations between SD and MMD. In clinical practice, we propose using MMD which is very simple to calculate, rather than SD. Regarding the incidence of proteinuria, the significance of this association between the variability of fasting TG and the incidence of proteinuria disappeared after adjusting for confounding factors. In our study, semi-quantitative proteinuria by urine dipstick was measured instead of 24 h urinary excretion or spot urinary protein/creatinine ratio. Although the dipstick method is simple and of low cost, quantitative evaluation of proteinuria is more accurate than the dipstick method [21]. The significance of the association between fasting TG variability and the incidence of proteinuria may be determined using quantitative evaluation of proteinuria.

Furthermore, it has been reported that individuals with high serum TG levels are more likely to develop CKD progression than those without [22]. Therefore, it is important to follow up serum TG level itself, which is easy to measure as a CKD risk factor in daily practice. However, even if we performed a Cox’s proportional hazard regression analysis adjusted by mean TG, the significance of the association between the fasting TG variability and eGFR decline remained (Table 2). This suggested that not only serum TG level itself, but also fasting TG variability were associated with eGFR decline. It is important to pay attention to serum TG itself, but monitoring fasting TG variability is also important as another risk factor. However, while a device for measuring the trend in 24-h blood pressure and plasma glucose level has been developed, no such device is available for the serum TG concentration. Therefore, it is necessary to consider how we can apply TG variability in daily practice.

In our study, the TG variability is year-to-year variability in TG measurements during fasting, which may differ from the risk of short-term intraindividual TG variability. Higher year-to-year variability of fasting TG may be a marker of incomplete or intermittent compliance with lifestyle measures such as changes in nutritional status, significant weight changes, or major changes in life circumstances. The risk of kidney events tended to be decreased by multifactorial intensive treatment, including lipid control in addition to control of the glucose level and blood pressure [23, 24]. Given these observations, it may be wise to include care for fasting TG variability in lipid control efforts.

The present study and our previous study have some common and some different aspects [13]. Firstly, in the present study, all the participants were quite healthy people who took medical checkups, while all the participants in the previous study were selected as diabetic kidney disease patients. Secondly, in this study, semi-quantitative proteinuria by urine dipstick was measured, while urine albumin-to-creatinine ratio was measured in the previous study. By using quantitative evaluation as in the previous paper, it may clarify the association between TG variability and the incidence of proteinuria. Lastly, our TG variability adopted fasting TG; however, postprandial TG variability was analyzed in the previous study. Usually, postprandial TG variability fluctuates more than fasting TG variability. The present study suggested that even fasting TG variability is associated with a prognostic risk factor in CKD. On the other hand, as a common point, higher TG variability was significantly associated with eGFR decline in both papers. In addition, higher TG variability was significantly associated with early incidence of albuminuria and proteinuria.

In a recently reported cohort study of patients with CKD, fenofibrate treatment protected against incident renal function worsening [25]. Potential nephroprotective mechanisms involve attenuated oxidative stress, inflammation, and apoptosis [26]. Particularly, pemafibrate, a selective PPARα modulator, has been in the spotlight due to its ability to selectively, potently, and safely interact with PPARα compared to other fibrates [27, 28]. Our study is not an interventional study; however, fibrates or the selective PPARα modulator may decrease TG variability which leads to protect against CKD progression.

This study has several limitations. Firstly, the participants were relatively healthy 40- to 75-year-old people who live in Okayama prefecture, and therefore selection bias was unavoidable. Secondly, this was a retrospective and observational study. Therefore, this study cannot state that lowering fasting TG variability prevents the progression of CKD. Thirdly, renal function possibly fluctuates depending on the seasons in Japan, and medical checkups are usually held between June and December. Lastly, the observational term of this study is relatively short; therefore, longer observation is required to clarify the association between TG variability and CKD progression.

## Conclusion

In conclusion, SD and MMD as the fasting TG variability may be a risk factor for eGFR decline and incidence of proteinuria in the participants who went through medical checkups. Further study will be warranted to clarify the association between TG variability and CKD progression.

## Supplementary Information

Below is the link to the electronic supplementary material.

Supplementary file1 (DOCX 106 KB)
