# Supplementary material for: The association of fasting triglyceride variability with renal dysfunction and proteinuria in medical checkup participants
Source: Clin Exp Nephrol. 2025 Feb 28;29(7):920–7. doi: 10.1007/s10157-025-02640-9 (PMC12205014; doi:10.1007/s10157-025-02640-9)
Supplement: Supplementary file 1 — Supplementary file1 (DOCX 106 KB) [file 10157_2025_2640_MOESM1_ESM.docx]

Supplementary Figure S1. The association between mean TG and fasting TG in 2017.


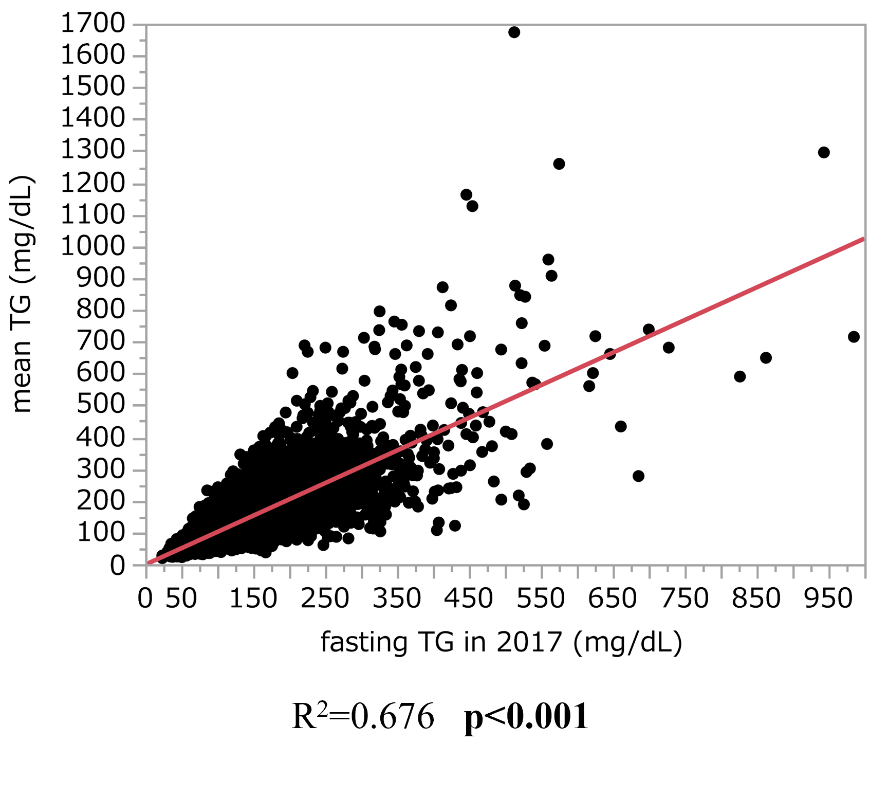


TG: triglyceride.

Supplementary Table S1. Multivariate Cox’s proportional hazard regression model for the association of fasting TG variability with incidence of proteinuria. (mean TG is excluded in the adjusting factors)

| HR [95% CI] p value | SD≥22 | MMD≥53 |
| --- | --- | --- |
| Model 1 | 1.08 [1.01-1.15] **0.0151** | 1.08 [1.02-1.15] **0.0129** |
| Model 2 | 1.08 [1.02-1.15] **0.0144** | 1.08 [1.02-1.15] **0.0119** |
| Model 3 | 1.06 [1.00-1.13] 0.0558 | 1.06 [1.00-1.13] **0.0479** |

Model 1: Adjusted for age, sex, and BMI

Model 2: Adjusted for age, sex, BMI, and baseline eGFR

Model 3: Adjusted for age, sex, BMI, baseline eGFR, smoking, DM, hypertension, antilipidemic drug intake, and alcohol consumption

TG, triglyceride; SD, standard deviation; MMD, maximum minus minimum difference; HR, hazard ratio; 95% CI, 95% confidence intervals; BMI, body mass index; eGFR, estimated glomerular filtration rate; DM, diabetes mellitus.
